# Cognitive Decline Assessment: A Review From Medical Imaging Perspective

**DOI:** 10.3389/fnagi.2021.704661

**Published:** 2021-08-18

**Authors:** Caroline Machado Dartora, Wyllians Vendramini Borelli, Michel Koole, Ana Maria Marques da Silva

**Affiliations:** ^1^School of Medicine, Pontifical Catholic University of Rio Grande do Sul, PUCRS, Porto Alegre, Brazil; ^2^Neurology Department, Hospital de Clínicas de Porto Alegre, Porto Alegre, Brazil; ^3^Brain Institute of Rio Grande do Sul, BraIns, Porto Alegre, Brazil; ^4^Nuclear Medicine and Molecular Imaging, Department of Imaging and Pathology, KU Leuven, Leuven, Belgium; ^5^Medical Image Computing Laboratory, School of Technology, Pontifical Catholic University of Rio Grande do Sul, PUCRS, Porto Alegre, Brazil

**Keywords:** brain imaging, aging brain, cognitive aging, Alzheimer's disease, PET, MRI

## Abstract

Aging is a complex process that involves changes at both molecular and morphological levels. However, our understanding of how aging affects brain anatomy and function is still poor. In addition, numerous biomarkers and imaging markers, usually associated with neurodegenerative diseases such as Alzheimer's disease (AD), have been clinically used to study cognitive decline. However, the path of cognitive decline from healthy aging to a mild cognitive impairment (MCI) stage has been studied only marginally. This review presents aspects of cognitive decline assessment based on the imaging differences between individuals cognitively unimpaired and in the decline spectrum. Furthermore, we discuss the relationship between imaging markers and the change in their patterns with aging by using neuropsychological tests. Our goal is to delineate how aging has been studied by using medical imaging tools and further explore the aging brain and cognitive decline. We find no consensus among the biomarkers to assess the cognitive decline and its relationship with the cognitive decline trajectory. Brain glucose hypometabolism was found to be directly related to aging and indirectly to cognitive decline. We still need to understand how to quantify an expected hypometabolism during cognitive decline during aging. The Aβ burden should be longitudinally studied to achieve a better consensus on its association with changes in the brain and cognition decline with aging. There exists a lack of standardization of imaging markers that highlight the need for their further improvement. In conclusion, we argue that there is a lot to investigate and understand cognitive decline better and seek a window for a suitable and effective treatment strategy.

## Introduction

Aging is associated with several transformations in our body, including the brain. The aging process causes modifications from molecular to morphological levels, thereby altering the brain size, vasculature, and, more often, cognition (Peters, 2006). However, biological and chronological aging is not completely linked. By 2050, the global life expectancy is expected to increase 6 years (the average global life expectancy is 72 years) (World Health Organization, 2017). Thus, it is necessary to understand how it will affect health, memory, and cognition of people. Aging influences both brain anatomy and function, but these phenomena are not well-understood. Oschwald et al. (2019) has emphasized the need to study the relationship between neuroanatomy and specific cognitive abilities in the aging brain.

Since the 1960s, cognitive decline has been diagnosed by using clinical signs (measured by tests and scores) and post-mortem evaluation of neurodegeneration and protein accumulation in the brain. In 1985, the *Archives of Neurology* published the first recommended use of neuroimaging, biomarkers, genetics, longitudinal studies, brain banks, and the establishment of family registries and animal models to study the phenomenon of normal brain aging (Khachaturian, 2005). New technologies have emerged in the field of diagnosis, treatment, care, and knowledge related to the causes of normal cognitive decline and Alzheimer's disease (AD). For example, diagnosis of AD involves conducting genetic tests for alleles of apolipoprotein ε (APOE ε), blood/spinal fluid test, amyloid-β (Aβ), and tau protein quantification and aggregation distribution by using positron emission tomography (PET). In the past decade, imaging biomarkers, including hippocampal volume in structural magnetic resonance images (MRI), temporoparietal glucose hypometabolism, neocortical Aβ, and medial temporal and neocortical tau deposition in PET images have been used to stage AD and understand the associated cognitive decline (Besson et al., 2015; Jack et al., 2018). However, which characteristics in PET and MRI indicate a prospective cognitive decline in the elderly population still need to be discerned.

Alzheimer's disease is a progressive, irreversible, and neurodegenerative disease that affects several regions of the brain, including the brain cortex and hippocampus (Citron, 2010; Masters et al., 2015). AD is associated with a dysfunction of the amyloid precursor protein (APP), leading to an accumulation of insoluble Aβ and generating plaques in extracellular spaces. The patients with AD present an inherent failure of the cerebral system to remove Aβ peptides (Masters et al., 2015). The amyloid cascade hypothesis suggests that Aβ super-production and failure in peptide clearance lead to amyloid deposition, triggering the production of neurofibrillary tangles (NFTs), cell death, synaptic loss, and symptoms of memory loss and cognitive decline (Cohen et al., 2012). In addition, AD is related to NFTs formed by the abnormal accumulation of hyperphosphorylated tau protein filaments (Masters et al., 2015). AD is associated with a significant loss of neurons and a deficit in the neuronal transmission system in brain areas related to memory and cognition, prominent inflammatory pathways, and innate immune response (Citron, 2010; Cohen et al., 2012).

Diagnosis of AD by using only clinical factors is often challenging; it can be misdiagnosed due to similarities in symptoms and biomarkers with other conditions, such as cerebrovascular diseases, dementia with Lewy bodies, frontotemporal dementia, and hippocampal sclerosis (Masters et al., 2015). In 2011, the National Institute on Aging–Alzheimer's Association (NIA–AA) workgroup revised the 1984 criteria for AD dementia by implementing guidelines and diagnostic criteria for neuropsychological testing, advanced imaging, and cerebrospinal fluid (CSF) measures, which could be used by both specialists with these tools available and general healthcare providers with no access to these tools (FDA-NIH Biomarker Working Group, 2016). The imaging biomarkers defined by NIA–AA include a decrease in the uptake of ^18^F-FDG in the temporoparietal cortex, a positive PET Aβ imaging, and atrophy in medial, basal, and lateral temporal lobes and medial parietal cortex detected by structural MRI.

In 2018, Jack et al. (2018) presented a research framework of NIA–AA with a biological definition of AD as an aggregate of neuropathological changes determined by *in vivo* biomarkers and post-mortem evaluation without considering clinical symptoms. It has proved beneficial in approximately 10– 30% of individuals who are clinically diagnosed with AD (demented individuals) but do not present neuropathological changes at autopsy and 30–40% of cognitively unimpaired (CU) elderly individuals who present with neuropathological changes in autopsy (Jack et al., 2018). Subjects who present amyloid and tau markers are defined as AD, and when only amyloids are present, individuals are known to have so-called Alzheimer's pathological change. This classification is based on pathological examinations and not clinical symptoms or the presence of neurodegeneration. Clinical symptoms without any biomarker evaluation are categorized as “Alzheimer's clinical syndrome” (ACS) and AD biomarker confirmation is used for staging the progression of the disease. Because the NIA–AA research framework was published in 2018 and is still being implemented, individuals denominated as “AD” or “probable AD” in this review are renamed ACS.

A few studies have analyzed the cognitive-decline images with the so-called AD-signature regions of interest (ROIs) that are brain regions that show remarkable changes in ACS. They comprise hippocampus in MRI and temporoparietal cortex and posterior cingulate cortex in ^18^F-FDG studies. However, with the new NIA–AA research framework proposed by Jack et al. (2018), these AD-signature ROIs have become invalid, because AD is pathologically defined as a proteinopathy that is characterized by the presence of amyloid and tau aggregates, not by hippocampus shrinkage or brain hypometabolism. The major limitation of this signature is that it cannot be used when a subject already has the imaging marker, and cognitive decline usually occurs in a stage where the pharmacological treatment for symptom retardation is unsuccessful. Consequently, it is essential to find early or set markers and their thresholds for healthy brain aging and the development of cognitive decline.

The present study addresses the following questions: Which biomarkers are used in cognitive decline assessment? How do dementia imaging patterns correlate with cognitive decline trajectories? How do brain glucose metabolism and amyloid and tau burden change with aging? How are cutoff values for classifying cognitive decline trajectories defined? How can joint evaluation of imaging biomarkers add value to the cognitive decline assessment? What are the trends and open questions in the assessment of cognitive decline that use medical images?

This review presents different views and aspects of cognitive decline evaluation by using medical images, primarily based on the differences between studies on CU individuals and those with cognitive declines, such as mild cognitive impairment (MCI) and AD. Biomarkers, including imaging markers, patterns based on ROIs, and their relationship with aging markers and neuropsychological tests have been discussed. Our goal is to delineate how aging has been studied with medical imaging and find answers to the above questions in the context of the aging brain and its cognitive decline.

## Biomarkers in the Cognitive Decline Assessment

A biomarker is an indicator of normal biological or pathogenic processes or responses to an exposure or intervention, including therapeutic interventions. Biomarkers can have molecular, histologic, radiographic, and physiologic characteristics with a direct effect, for example, measurement of amyloid, or indirect effect such as ^18^F-FDG imaging to measure neuronal activity. Furthermore, biomarkers are divided into different categories such as predictive, prognostic, diagnostic, response seeking, monitoring, safety, and risk (FDA-NIH Biomarker Working Group, 2016).

Aging biomarkers are the indicators of the functional state of a person and the risk factors for specific age-related pathologies; these include specific proteins in the CSF, brain structural images, and pathological proteins (Moskalev, 2019). Prognostic biomarkers can be used in clinical trials to screen patients with a high risk of having a disease-related endpoint event or a substantial worsening. Several markers have been used to study the decline in human brain activity, with cognitive tests being the gold standard. Both direct and indirect imaging markers have shown promising results in differentiating brain patterns in the early stages of decline.

A strict relationship exists between physiological biomarkers and imaging markers. Physiological biomarkers are measurable physical aspects such as a clinical symptom or blood glucose, which are evaluated by their values in normal biological or pathogenic processes (Strimbu and Tavel, 2010). Imaging (bio)markers are characteristics visualized by analyzing a medical image.

Amyloid PET image detects amyloid plaques and is based on the staining agents used in post-mortem studies. In 2004, the first-generation Aβ tracer, the Pittsburg compound B (^11^C) or ^11^C-PiB, was developed for *in vivo* evaluation of Aβ plaque accumulation as it was only possible in a post-mortem examination. The compound is derived from a staining agent called thioflavin-T and is similar to ^18^F-NAV4694, a third-generation agent. Another PET Aβ tracer is florbetapir (^18^F) or ^18^F-AV45 that is derived from Congo red and Chrysamine-G. All PET Aβ tracers bind to fibrillary forms of Aβ, mostly Aβ42 fibrils. The differences among different PET Aβ radiotracers are attributed to the specific binding on Aβ plaques, uptake time, and clearance (Bao et al., 2017).

Recently, several tau PET tracers, such as ^18^F-AV1451, ^18^F-T808, ^18^F-THK5351, and ^11^C-PBB3, have been developed. However, each one of these has different binding properties with tau isoforms. For example, ^18^F-AV1451 shows a high affinity with classical, paired helical filament–tau tangles in its six isoforms and low affinity with tangles of the 4R isoforms. ^18^F-THK5351 exhibits a high signal-to-background ratio and binding affinity for hippocampal damaged tissue but off-target binding for MAO-B. Similarly, ^11^C-PBB3 has a high specificity for tau deposition; however, its sulfate conjugate crosses the blood–brain barrier, hindering the quantitative evaluation of the tau tracer (Bao et al., 2017). The tau PET brain pattern distribution at different disease stages can be explained by six different Braak stages (Braak and Braak, 1991) that are based on post-mortem evaluation of NFTs and neuropil threads (NTs). Stage I is represented by the modest involvement of the transentorhinal region, a few isolated NFTs in the regions of the basal forebrain and thalamus. Stage II is an aggravation of stage I with hippocampal involvement and isolated NFTs in isocortical regions. Stage III consists of a severe attachment of NFTs in entorhinal and transentorhinal regions; mild involvement of the hippocampus and isocortex, forebrain nuclei, thalamus, and amygdala, scattered NFTs and NTs in the basal portions of frontal, temporal, and occipital areas and subiculum. Stage IV is characterized by a thalamic nucleus densely filled with NFTs and NTs. In stage V, the isocortex is severely affected and the thalamus, claustrum, and amygdala are more consistently involved. In stage VI, all stage IV changes are more pronounced with a considerable loss of nerve cells and all isocortical regions, such as severely affected subcortical nuclei (Braak and Braak, 1991).

In summary, relevant regions for different Braak stages are as follows (Braak and Braak, 1991; Alafuzoff et al., 2008):

- Braak I: transentorhinal region.
- Braak II: entorhinal region.
- Braak III: temporo-occipital gyrus.
- Braak IV: temporal cortex.
- Braak V: peristriatal cortex.
- Braak VI: isocortical areas, subcortical nuclei, and extrapyramidal system (striatal cortex).

Analyzing different image markers together can be challenging. A voxel-wise analysis (Besson et al., 2015) found that the brain patterns of healthy elders selected independently as positive or negative for biomarkers (metabolism, degeneration, or amyloid burden) did not match with the patterns found in a group with positive or negative subjects for another biomarker of the same list. Healthy elders with higher hypometabolism showed a global distribution of hypometabolic areas, especially in the frontal cortex. The prevalence of amyloid positivity increased from 10 to 44% in CU subjects aged from 50 to 90 years (Ewers et al., 2012; Besson et al., 2015; Jansen et al., 2015).

In a study by Jack et al. (2019), the Aβ/tau/neurodegeneration, AT(N), system showed a significant improvement in predicting memory decline in non-demented elders. The AT(N) system was proposed in the NIA–AA research framework (Jack et al., 2018) to categorize elder individuals by pathology. The AT(N) system was defined by several biomarkers, where A represented amyloid markers, T represented tau, and N represented the presence of neurodegeneration (or neuronal injury, atrophy on MRI, FDG hypometabolism, and CSF total tau). The presence of A and T positives are neuropathological indicators of AD, whereas (N) is not a disease specific (Jack et al., 2018, 2019) enabling the use of different measures with similar but not completely redundant information (Jack et al., 2015). In non-demented elders, Jack et al. (2019) showed that individuals with A+T–(N)+ showed cognitive decline at all ages, independent of APOE ε4 presence, whereas the cognitive decline in A+T–(N)– individuals was slower than in other evaluated groups with a positive amyloid biomarker.

Memory scores and amyloid burden have been extensively studied. Chételat et al. (2011) Click or tap here to enter text. assessed the correlation between Aβ deposition and episodic memory scores. After reviewing the literature, they found that pooling the subjects in groups with different decline patterns (CU elders, MCI, and ACS) could drive erroneous correlations. For example, ACS and non-demented individuals, when pooled together in an Aβ deposition analysis and memory test, showed a high correlation due to a higher level of Aβ deposition in ACS and not due to the whole group representation. However, subjects in predementia stages had lower episodic memory performances due to the Aβ deposition, especially in the temporal neocortex, and independently because of hippocampal atrophy. Ewers et al. (2012) suggested that the first step in predicting cognitive decline is assessing the combination of structural and functional brain decline associated with Aβ deposition. Although amyloid accumulation has been repeatedly associated with further memory decline in longitudinal studies (Lim et al., 2014; Farrell et al., 2017; Landau et al., 2018), high amyloid accumulation, including intermediate “gray zone” burden (Ebenau et al., 2020), is associated with further memory decline (Landau et al., 2018), but not baseline amyloid levels. Amyloid accumulation across multiple posterior regions predicted memory decline (Farrell et al., 2018), but a specific region within the superior temporal sulcus of CU individuals was associated with memory decline (Guo et al., 2020).

The problem of wrong correlation between the instrumental activities of daily living (IADL) and tau and Aβ burden occurs during pooling the groups with different decline patterns. MCI and ACS groups, when pooled, showed higher tau and Aβ uptake than CU individuals, making the correlation with IADL stronger. When analyzed individually, the association between tau and amyloid burden and IADL impairment was weak (Halawa et al., 2019). Although the evidence suggested an emerging heterogeneity of biomarker expression in ACS subjects (Osorio et al., 2014) as the AT(N) system, still there are no standard cutoff values for evaluating the biomarkers.

High Aβ accelerates atrophy in CU elders in the medial temporal lobe and precuneus compared with subjects with low Aβ levels (Ewers et al., 2012). Chételat et al. (2011) studied the effects of temporal Aβ deposition and found that it had no relation with memory and hippocampal atrophy. Recent studies have shown that disentangling the effects of Aβ and tau on cognitive decline is not an easy task. The accumulation of both proteins has a relationship with age in cognitively impaired and unimpaired individuals (Lowe et al., 2018). An increase in tau abnormality was associated with age in Aβ+ and Aβ− CU individuals and was not confined to the medial temporal lobe, being widespread through the brain, mostly corresponding to early Braak stages I–IV (Lowe et al., 2018; Pascoal et al., 2020). These isolated cases of tau pathology, without amyloid and neurodegeneration abnormal markers, occurred in the absence of cognitive impairment (Altomare et al., 2019). Cognitive decline has shown to be associated with abnormal tau levels, independent of Aβ levels; however, it was associated with increased worsening of memory when associated with abnormal Aβ (Sperling et al., 2019; Guo et al., 2021). The contrary was not confirmed: abnormal Aβ levels without abnormal tau are not related to cognitive decline in CU individuals (Sperling et al., 2019; Guo et al., 2021; Pereira et al., 2021). There is no consensus on which biomarkers can be used to assess cognitive decline and how they are associated. It is hard to find an agreement within the studies in evaluating the cognitively healthy older adult population.

## Imaging Biomarkers in Cognitive Decline Trajectories

Neurodegeneration, glucose hypometabolism patterns, amyloid, or tau burden are the primary characteristics of brain-imaging analysis. Clinical studies usually focus on analyzing (AD–) signature ROIs. It is used as a differential diagnosis for ACS. However, specific regions for analysis in Aβ and tau studies are not well-understood. In this section, we will present findings of each imaging biomarker (MRI and FDG, amyloid or tau PET) in cognitive-decline trajectory.

MRI have been widely used to evaluate the decline and differential diagnosis of ACS due to its high spatial resolution and structural characteristics. The most clinically used ROI in MRI is the hippocampus for shrinkage compared to CU elders. However, studies showed different structures with neuroanatomical changes in healthy aging. Ewers et al. (2012) measured the gray matter (GM) volume in regions such as the hippocampus, middle temporal gyrus, superior temporal gyrus, amygdala, parahippocampus, entorhinal cortex, inferior parietal lobe, precuneus, and thalamus. A meta-analysis (Schroeter et al., 2009) showed that these regions are more predictive of ACS than the hippocampus and associated MRI measures with the Aβ scale (Ewers et al., 2012). In addition, he found that MCI individuals had a more significant effect of Aβ on the annual rate of volume decline in the inferior parietal lobe, entorhinal cortex, parahippocampus, middle temporal gyrus, inferior parietal lobe, and a trend for the precuneus.

Besson et al. (2015) performed a voxel-wise analysis of CU individuals with positive and negative MRI biomarkers. He used the hippocampal volume as the ROI and found that CU individuals with a positive marker for atrophy showed lower executive-function performance than its counterpart (MRI-negative individuals). In addition, positive subjects showed a significantly lower volume in the hippocampus, frontoinsular, ventromedial, prefrontal, and lateral temporal cortex bilaterally. Rizk-Jackson et al. (2013) conducted a longitudinal 48-month study and found that the volume loss in the hippocampus, temporal lobe, and the overall brain was higher in elder subjects who experienced cognitive decline relative to those who remained stable.

Chételat et al. (2011) assessed the brain regions with a higher difference between ACS and CU individuals in the GM and white matter (WM) of T1-weighted MRI in a different voxel-wise approach. The regions were turned to a mask and assessed for a correlation between regions and episodic memory scores in healthy elders and MCI. They found that GM atrophy was mainly located in the hippocampus and temporal neocortex, extending to the temporoparietal, temporo-occipital, anterior cingulate cortex, and precuneus regions. WM atrophy involved the cingulum bundle, perforant path, and corpus callosum. The relationship with episodic memory scores and GM volume was confined bilaterally to the hippocampi, with no relation with the WM volume in family-wise error corrected threshold of *p* < 0.05. However, when the *p*_uncorrected_ < 0.001 was applied, a significant correlation was found in the perforant path bilaterally. The mean cortical thickness of entorhinal, inferior temporal, middle temporal, and fusiform regions was used to find cutoff values for GM degeneration for a marker to differentiate cognitively impaired individuals from unimpaired individuals (Jack et al., 2017a). MRI is a fundamental imaging modality in clinical practice, which provides useful information about the progression of cognitive decline in healthy older adults. When associated with amyloid positivity, MRI can strongly predict further decline (Jack et al., 2018). However, MRI findings presented mixed patterns in patients with consistent memory complaints, the theoretical first symptom of AD, which makes the utility of MRI in early AD neurobiology unclear (Wang et al., 2020).

Jie et al. (2015) used a selection feature method to find the most important brain regions in differentiating between MCI subjects and healthy elders. Volume (based on MRI) and the ^18^F-FDG average intensity of 93 brains ROIs were used. A manifold regularized multitask selection feature between MCI and healthy elders was applied. The selected brain regions were localized mostly on the left (L) brain side: L. cuneus, L. and right (R) precuneus, L. temporal pole, L. entorhinal cortex, L. and R. hippocampal formation, L. angular gyrus, L. and R. occipital pole, R. amygdala, L. parahippocampal gyrus.

Rizk-Jackson et al. (2013) used ^18^F-FDG images to determine which clinical measure could classify healthy elders who remained stable and those whose condition progressed to MCI. An ROI-based analysis calculated the average glucose metabolism in the right and left angular gyri, right and left temporal gyri, and bilateral posterior cingulate gyrus. An analysis of the differences between healthy elders and those who progressed to MCI revealed that only posterior cingulate cortex hypometabolism showed statistical significance, bringing back the idea of a signature ROI.

An ROI-based study on associations between Aβ levels and ^18^F-FDG uptake (Ewers et al., 2012) used a meta-analysis of regions typically affected in ACS, based on previous studies (Jagust et al., 2009; Landau et al., 2011). The selected areas for ^18^F-FDG analysis were the angular gyrus, posterior cingulate/precuneus, and inferior temporal cortex.

Besson et al. (2015) defined the ^18^F-FDG analysis regions by using the most remarkable changes in ACS areas in an independent sample. These regions were the posterior cingulate and temporoparietal, the AD-signature ROIs. In addition, he used a binary mask corresponding to the entire GM, except for the cerebellum, occipital, and sensory-motor cortices, hippocampi, amygdala, and basal nuclei to study an Aβ signature in a healthy elder (between 50 and 84 years of age) group.

By using the group of healthy elders (Besson et al., 2015), Oh et al. (2014) examined the regional patterns of Aβ deposition, glucose metabolism, and GM volume and their correlation with cognition using composite scores from neuropsychological tests. He calculated a global PIB index based on the mean distribution volume ratio values of large cortical ROIs that spanned through the frontal, temporal, and parietal cortices and anterior/posterior cingulate gyri. A correlation with Aβ topography using the scaled subprofile modeling analysis was found. In addition, reduced amyloid deposition in the hippocampus bilaterally and the visual and motor cortex was found. However, positive amyloid deposition was found in the medial frontal, temporoparietal, lateral cortices, and precuneus. A negative correlation was present between GM volume and global PIB index in the medial frontal, lateral temporal, and posterior cingulate cortices and hippocampus and positive loadings in the superior frontal, primary sensory/motor, and visual cortices. The relationship of global PIB index increased with a relative decrease in glucose metabolism in the inferior medial frontal cortex, lateral and medial temporal cortex, anterior cingulate, and visual cortex, and relative increase in the lateral prefrontal cortex, lateral parietal cortex, and precuneus.

Chételat et al. (2011) performed a voxel-wise analysis of Aβ images between ACS and CU individuals. Regions with higher differences between the groups were the posterior cingulate-precuneus area, anterior cingulate and medial frontal cortex, and lateral temporal and temporoparietal regions. They found a significant correlation between Aβ deposition and episodic memory scores in the inferior and middle temporal neocortex regions, anterior and posterior cingulate, and prefrontal cortex. Ewers et al. (2012) used ROIs for Aβ evaluation, comprising the prefrontal, lateral temporal, anterior cingulate gyrus, parietal, and posterior cingulate/precuneus area, the same regions as in a previous study (Halawa et al., 2019).

A pathological study comparing Aβ burden by immunohistochemistry and ^18^F-florbetapir uptake in ACS elders showed a good correlation with the frontal, temporal, parietal, anterior and posterior cingulate, and precuneus regions (Clark et al., 2011). These regions were used to analyze longitudinal changes in unimpaired older individuals and progression of the Aβ burden. However, the rate of Aβ accumulation was dependent on the reference region used to calculate the standardized uptake value (SUV) ratio (Landau et al., 2015). Moreover, these regions were not related to age, baseline memory, or executive function in longitudinal (Landau et al., 2018) and cross-sectional studies (Jansen et al., 2018), but they were associated with higher Aβ in baseline, poorer longitudinal memory performance (Landau et al., 2018), and CDR changes (Mormino et al., 2017) and contributed to the individual estimates of cognitive level in the transversal approach (Jansen et al., 2018). Furthermore, for MCI and dementia of uncertain etiology, the use of amyloid PET images has proved to be useful in challenging clinical diagnosis (Rabinovici et al., 2019). In contrast, cortical Aβ deposition did not affect cognitive and behavioral domains within 2 years in CU older individuals (70 years old or more) with subjective cognitive decline (Dubois et al., 2018).

Increased tau uptake in the meta-ROI can accurately distinguish AD dementia from other dementias (Ossenkoppele et al., 2018) with a variety of tau tracers (Leuzy et al., 2021). In addition, it can predict memory decline in cognitively healthy older adults (Jack et al., 2019). Despite its high accuracy for AD-related brain alterations, the potential use of tau PET in clinical practice remains to be thoroughly discussed. Interesting patterns were found in a voxel-wise analysis using Aβ and tau images (Shimada et al., 2017). Tau pathology showed a gradual expansion with age within a restricted region around the medial temporal cortex. A recent study suggested that brain amyloid accumulation may occur earlier than tau-related axonal damage (Pereira et al., 2021). Thus, in the presence of Aβ, tau progression occurred in the entire neocortex via the collateral sulcus. Medial temporal atrophy was a normal finding in healthy aging that was probably caused by tau pathology even without a significant association between tau burden and brain volume in the hippocampus.

Halawa et al. (2019) used the regions related to the IADL scores from previous studies (bilateral entorhinal cortex, inferior temporal cortex, rostral anterior cingulate cortex, posterior cingulate cortex, supramarginal gyrus, orbitofrontal cortex, precuneus, and dorsolateral prefrontal cortex) for tau imaging to investigate the association between IADL impairment and regional cerebral tau deposition in healthy elders, MCI, and ACS subjects. He performed the same analysis for Aβ images but used the frontal, cingulate, and lateral parietal and lateral temporal cortices. He found more significant medial and inferior temporal tau and cortical Aβ burden associated with greater IADL impairment.

**Figure 1:**
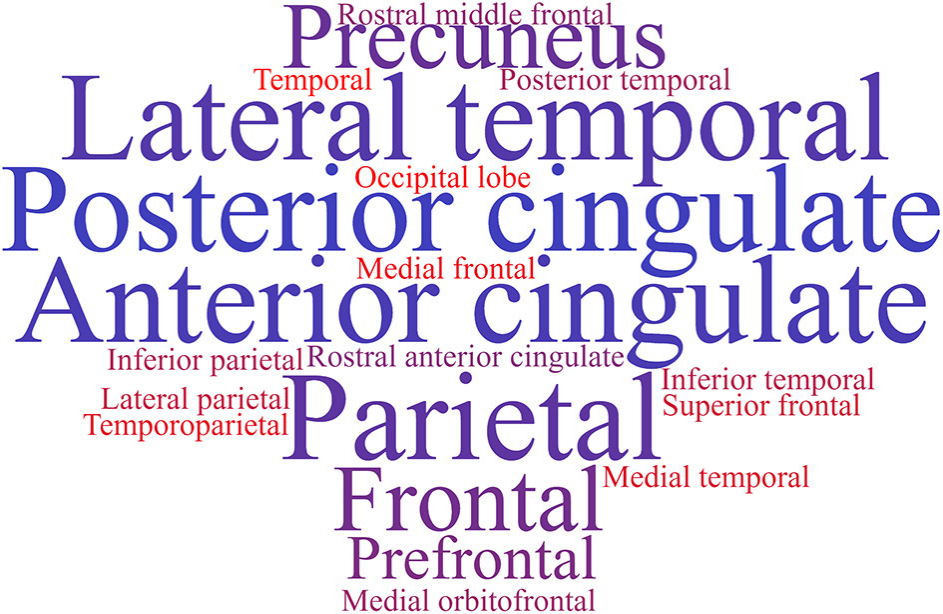
Tag cloud of the most used brain regions in amyloid studies based on the information retrieved from seven papers (Chételat et al., 2011; Ewers et al., 2012; Oh et al., 2014; Besson et al., 2015; Mattsson et al., 2015; Hanseeuw et al., 2017; Halawa et al., 2019).

The brain regions for analyzing the initial cognitive decline are not well-defined. Even the AD-signature ROIs are not the best alternative for the analysis because their characteristics are better represented when transitioning between MCI and ACS. The brain regions to be analyzed are still miscellaneous for MR, ^18^F-FDG, amyloid, or tau PET images and are usually related to the marker. Figure 1 shows an example of a tag cloud built with the most commonly diagnosed brain regions in amyloid PET studies, extracted from seven papers (Chételat et al., 2011; Ewers et al., 2012; Oh et al., 2014; Besson et al., 2015; Mattsson et al., 2015; Hanseeuw et al., 2017; Halawa et al., 2019).

## Aging and Brain Glucose Hypometabolism

Like brain atrophy, hypometabolism is a neurodegeneration signal detected with structural MRI. Hypometabolism, being common in aging, could predict cognitive decline. However, metabolism patterns are not always related to all aging image markers.

Besson et al. (2015) found that the ^18^F-FDG positive (FDG+) vs. negative (FDG–) group (with positive cutoff values defined in the AD-signature ROIs as the 90th percentile of the biomarker residuals estimated in an independent group of ACS subjects) did not reveal a typical AD-like pattern of decreased volume in MRI and an increased Aβ burden. However, they detected a mixed hypometabolic pattern, including AD-signature ROIs and the prefrontal cortex. The involvement of the prefrontal cortex may be related to the non-ACS process, but with healthy aging or frontotemporal dementia, because it is expected to appear in the later stages of ACS. Hypometabolism in the prefrontal cortex can be age related, and Aβ deposition may not be associated with degeneration. In their findings, no statistical differences were found in age, sex, education level, or APOE ε4 between FDG+ and FDG– subjects (Besson et al., 2015).

Ishibashi et al. (2018) studied the effect of aging on brain glucose metabolism and analyzed CU elderly individuals longitudinally (67.9 and 75.7, mean age at baseline and second scan, respectively). The analysis was voxel-based and showed a decrease in the ^18^F-FDG uptake in the anterior cingulate, posterior cingulate/precuneus, and lateral parietal cortices in healthy aging. However, the Mini-Mental State Examination of these subjects remained CU (29.2 ± 1.1, ranging from 25 to 30) in the time point of the second scan showing that it is not directly related to cognitive decline even with the glucose metabolism decrease.

Oh et al. (2014) found that ^18^F-FDG patterns did not account for individual differences in cognition to the spectrum of healthy control aging. Healthy elders presented a relative increase in glucose metabolism and Aβ deposition in the posterior cingulate/precuneus and lateral parietal and prefrontal cortices. Hypometabolic regions in brain glucose metabolism images did not show a direct relation to aging. Although the brain hypometabolism in temporoparietal regions of the brain was commonly used as a marker of cognitive decline, it was not related to cognitive decline but to a normal hypometabolism due to age.

## Aging and Amyloid Burden

Neurodegeneration biomarkers include morphological and metabolic measures, such as hippocampal atrophy and posterior cortical glucose metabolism (Wirth et al., 2013). The relationship between brain hypometabolism and the amyloid burden was interpreted as evidence of disruption of neuronal functions and synaptic activity (Oh et al., 2014). However, Oh et al. (2014) showed that both higher levels of Aβ and relative increase in glucose metabolism were present in a population of healthy elders. Besson et al. (2015) found similar results, with subjects with reduced brain glucose metabolism in (AD–)signature ROIs without a high Aβ deposition. Conversely, Wirth et al. (2013) did not find significant associations between amyloid tracer uptake, cortical hypometabolism, and hippocampal atrophy. Brain hypometabolism and Aβ burden are unrelated. The two markers showed that Aβ might induce neurodegeneration with a temporal delay, with a relation of additivity instead of sequentially in the decline process.

Oh et al. (2014) hypothesized that no correlation between brain hypometabolism and Aβ burden could be attributed to several factors. One of them was related to the microglia surrounding the Aβ plaques, producing an inflammatory reaction that may increase the glucose metabolism in these regions. Other hypotheses are based on the possibility of an increase in abnormally hyperactive neurons in cortical circuits, alterations in brain homeostasis, and increased neural activity due to Aβ production. The cognitive reserve and its involvement in brain aging are also other hypotheses for maintaining cognition even with deposits of Aβ.

Cognitive reserve was proposed due to the differences between brain damage and pathology (observed in imaging markers) and its clinical manifestations (Stern, 2009). It is postulated that individual differences in cognitive processing and task performance allow certain individuals to cope better with brain damage (Stern, 2009). Due to this coping mechanism, some subjects could have elevated brain metabolism even with amyloid deposition (Ewers et al., 2012). However, it is unclear how to measure cognitive reserve from a neurobiological view (Stern, 2009). An increase in brain glucose metabolism in Aβ-accumulated regions could be a natural compensatory mechanism, permitting elders to remain CU even with Aβ plaques. Thus, a longitudinal analysis of the behavior of brain metabolism and Aβ features needs to be jointly evaluated (Oh et al., 2014). Other features that need to be assessed extensively are the relationship between cognitive domains and Aβ burden.

Episodic memory and executive functions are two cognitive domains that decline with advancing age (Oh et al., 2012). However, there is an unclear relationship between episodic memory, executive domains, and imaging biomarkers. Wirth et al. (2013) found that the Aβ burden was related to longitudinal decline instead of cross-sectional cognitive decline. A similar study (Jang et al., 2019) showed that Aβ+ MCI individuals who showed cognitive decline within 3 years to ACS had a higher Aβ burden than those with a slow decline (after 3 years). In addition, Aβ- MCI has a considerably lower chance of dementia conversion in 3 years than Aβ+ MCI. Moreover, Wirth et al. (2013) found that hippocampal neurodegeneration biomarkers do not significantly interact with Aβ uptake status on the longitudinal executive function decline. Jang et al. (2019) showed that additional abnormal neurodegeneration markers worsened the prognosis in Aβ+ MCI individuals within 3 years.

Mattsson et al. (2015) evaluated the amyloid tracer ^11^C-AZD2184 binding in elder subjects (between 58 and 71 years old) with and without a decline in episodic memory. They hypothesized that the Aβ burden was more common in subjects with decline. On the contrary, the amyloid binding was higher in healthy elders than in those with a decline. Wang et al. (2020) reported no agreement between amyloid load and cognitive decline in the characterization of subjective cognitive decline.

Nebes et al. (2013) evaluated healthy elders using amyloid PET scans and cognitive tests and divided the subjects into Aβ-positive and Aβ-negative individuals. No differences were found between the groups and a set of cognitive scores (including tests for information processing speed, working memory, and inhibitory control). The only difference was that Aβ-positive subjects had a higher frequency of APOE ε4 carriers than Aβ-negative subjects. Wirth et al. (2013) found a correlation between Aβ positivity and a decline in semantic and visual memory and visuospatial abilities. The amyloid burden did not affect logical memory and executive functions. Jessen et al. (2020) showed that CU individuals with subjective cognitive decline and Alzheimer's disease biomarkers without objective cognitive impairment presented a 40–62% increased chance to progress to MCI or AD within 3 years.

Oh et al. (2012) found that although limited to visual domains in cognitively healthy elders, visual episodic memory is negatively associated with Aβ deposition and the degree of covariance pattern of Aβ deposition. According to Oh et al. (2012), the difference could be related to cognitive reserve, because elders with higher cognitive reserves showed no correlation between Aβ burden and decline in cognitive performance, obscuring an Aβ effect. However, Oh et al. (2014) found that the GM volume Aβ-dependent patterns did not account for individual differences in cognition in the spectrum of healthy aging.

Longitudinal studies revealed the fastest decline in Aβ deposition. Healthy elders with a high amyloid burden have a higher tendency to progress to MCI within 3 years (Rizk-Jackson et al., 2013), and the interaction of Aβ pathology with neurodegenerative biomarkers could exacerbate cognitive worsening (Wirth et al., 2013). The relationship between brain hypometabolism, atrophy, and Aβ burden is not well-defined. These processes appear to be more additive than sequential in aging and cognitive decline and can be associated with other brain changes, such as microglia activation or alterations in brain homeostasis. In addition, the relationship between cognitive domain performance and the Aβ burden is not well-understood. More longitudinal studies are required for a better panorama on the changes in the brain and cognition with aging in both cases.

## Aging and Tau Burden

Recently, new phospho-tau radiotracers have allowed the study of cognitive aging trajectories. The deposition of phospho-tau (p-tau) in the brain of CU individuals is an inevitable consequence of the aging process (Braak et al., 2011), following a specific neuropathological sequence (Braak's stages). Braak's stages are closely related to memory dysfunction, also reflecting the disease progression (Bao et al., 2021). The increased p-tau burden in cognitively healthy older adults must be carefully interpreted (Castellani, 2020).

Although primary age-related tauopathy (PART) has been described in both pathological and imaging studies, it is poorly related to clinical symptoms (Crary et al., 2014; Harrison et al., 2019). The accumulation of tau in healthy older adults spreads to the bilateral temporal lobe and retrosplenial regions (Harrison et al., 2019) and increases with age. In CU older adults, medial temporal tau deposition is related to memory decline, associated with decreased volume in these regions (Ziontz et al., 2019). A temporal meta-ROI accumulation was proposed to be highly specific for AD tau brain pathology and is uncommon in CU older adults (Ossenkoppele et al., 2021a). Furthermore, the temporal meta-ROI has been used with different tau-PET tracers (Leuzy et al., 2019) and in different cognitive aging trajectories, reflecting Braak stages I to IV (bilateral entorhinal, amygdala, fusiform, inferior and middle temporal cortices, respectively) (Ossenkoppele et al., 2018; Pereira et al., 2021).

The accumulation of tau was higher in CU individuals with imaging and clinical variables consistent with AD, such as amyloid positivity and baseline cognitive performance (Jack et al., 2020). Amyloid positivity is a strong predictor of temporal tau accumulation in CU individuals (Jack et al., 2020). Tau accumulation is magnified by amyloid deposition, especially in individuals with MCI or AD dementia (Smith et al., 2020). However, young, amyloid-positive individuals show an accelerated rate of tau deposition. Lower baseline cognitive performance is associated with higher tau deposition in the temporal lobe (Pontecorvo et al., 2017) and neocortical regions (Maass et al., 2017; Ziontz et al., 2019). Compared to amyloid PET and MRI, tau PET has emerged as the most promising tool for predicting cognitive change in Aβ+ individuals (Ossenkoppele et al., 2021b).

## Classifying Cognitive Decline Trajectories Using Imaging Biomarkers

For each biomarker, a different cutoff value was used for defining its positive or negative presence. According to Mckhann et al. (2011), biomarker results are normal or abnormal, positive or negative in several cases, and a qualitative interpretation is enough. However, the problem of ambiguous or indeterminate results exists because biomarkers have a continuous measure, and cutoff values are applied to continuous biological phenomena. Furthermore, quantitative and objective image analysis may not completely resolve the issue of the lack of standard values to differentiate normal and abnormal biomarkers.

Ewers et al. (2012) studied the association of Aβ PET and CSF (Aβ_1−42_) in healthy elders and MCI subjects in a 2-year rate of cognition change based on memory and cognitive scores, regional GM volume (hippocampus, middle temporal gyrus, superior temporal gyrus, amygdala, parahippocampus, entorhinal cortex, inferior parietal lobe, precuneus, and thalamus), and brain metabolism assessed with ^18^F-FDG (in the bilateral angular gyrus, posterior cingulate/precuneus, and inferior temporal cortex). He used a cutoff value of 1.6 to define an amyloid group dichotomization. When 1.5 and 1.41 cutoff values were applied to other studies, different results were found in Aβ patterns, with no difference in the cognition change rate. Previous studies showed different cutoff values depending on the parameters used for Aβ evaluation and discrimination on its presence (positive) or absence (negative). Table 1 shows certain cutoff values and parameters used in the literature.

**Table 1:** Cutoff values for Aβ tracers from the literature.

**References**	**Cutoff value**	**Additional information**	**Cohort data**	**Diagnostic performance**
Besson et al., 2015	1.005	90th percentile of estimated values in a group of 26 CU individuals aged 31 ± 8.4 years from the IMAP project.	54 CU elders between 50 to 84 years old (mean age 65.8 ± 8.3) recruited from the community	15% of CU individuals were Aβ+.
Ewers et al., 2012	1.6	Minimum density value of ^11^C-PiB PET scores between the 2 modes of the probability density function of mean ^11^C-PIB scores of 19 CU, 65 MCI, and 19 ACS individuals^*^.	124 CU, 229 amnestic MCI, and 112 ACS individuals from ADNI that have ^11^C-PiB or CSF Aβ_42_ data.	iPIB+ was present in 92.0% of the ACS, 72.5% of the MCI, and 41.1% of the CU subjects.
Halawa et al., 2019	1.4	SUV ratio determined based on the distribution across the entire sample.	51 CU, 39 MCI/AD individuals from ADNI with a mean age of 76.3 ± 6.9 years;	22.9% of CU, 40% of MCI, and 100% of ACS individuals were Aβ+.
Hanseeuw et al., 2017	1.34	Gaussian mixture model of the DVR of 277 CU individuals from the HABS.	Two samples of CU individuals from HABS according to the availability of tau imaging (90 CU) or memory follow-up (277 CU).	First sample: 36.6% of CU were Aβ+. Second sample: 28.5% of CU were Aβ+.
Jack et al., 2017b	1.42	SUV ratio based on the reliable worsening cutoff method. It is equivalent to a Centiloid value of 19.	CU, MCI, and ACS individuals between 30 to 95 years old of the Mayo Clinic Study of Aging (MCSA).	71% of autopsied individuals with Thal phase <2 were Aβ-; 92% with Thal phase > 2 were Aβ+.
Maass et al., 2018	1.065	Calculated using the DVR of previous literature.	83 CU from the BACS with a mean age of 77 ± 6 years.	56.6% CU were classified as Aβ-, and 43.4% CU as Aβ+.
Nebes et al., 2013	1.50 to 1.78	Calculated on the anterior cingulate, anterior-ventral striatum, precuneus, frontal, lateral temporal, and parietal cortex, of 62 CU individuals.	71 CU between 65 and 88 years, and 37 younger individuals between 18 and 30 years, recruited from the community.	25% of CU individuals were Aβ+, 75% were Aβ-.
Oh et al., 2011	1.08	Mean DVR of young (±25 years) and elders (+65 years) + 2 standard deviations of young adults within the frontal, temporal, parietal, and anterior/posterior cingulate regions.	52 CU with a mean age of 74.1 ± 6.0 years recruited from the community.	36.5% of CU elders individuals were classified as Aβ+, and 63.5% as Aβ-.
Shimada et al., 2017	1.34	Mean cortical SUV ratio which maximizes the sum of sensitivity and specificity for discrimination between CU and AD individuals.	10 young CU (38.2 ± 4.7 years) and 18 older CU (67.3 ± 6.4 years), volunteers from the National Institute of Radiological Sciences, and 9 MCI (74.2 ± 4.4) and 17 ACS (71.6 ± 9.6) individuals from the Chiba University Hospital.	Young CU were considered Aβ. Study design excluded older CU individuals that were Aβ+. All MCI and ACS individuals were Aβ+.
Wirth et al., 2013	1.08	Mean Aβ uptake + 2 standard deviations of the frontal, temporal, parietal, and anterior/posterior cingulate regions derived from an independent group of healthy young adults.	38 CU individuals recruited from the BACS between 61 and 87 years.	65.8% of individuals were Aβ-, and 34.2% were Aβ+.

* *iPIB model was created for these subjects that did not have ^11^C-PIB scans and was calculated using least square regressions to estimate PIB score based on the correlation of CSF Aβ_1−42_ and Apolipoprotein E ε4 genotype with ^11^C-PIB. BACS, Barkeley Aging Cohort Study; DVR, distribution volume ratio; HABS, Harvard Aging Brain Study; SUV, standardized uptake value*.

The Centiloid project aims to produce comparable methods across imaging centers to analyze amyloid PET images and solve the problem of applying a universal cutoff value between normal and abnormal ranges of amyloid deposits. It uses a linear scale for data of any amyloid PET image to an ^11^C-PiB-based scale. The scale has an average value of zero for “high-certainty” amyloid-negative subjects and a value of 100 for typical AD subjects. Images in Centiloid units are interpretable longitudinally and across several imaging centers by using ^11^C-and ^18^F-amyloid tracers (Klunk et al., 2015).

In 2017, Jack et al. (2017b) developed and defined cutoffs for amyloid PET, FDG PET, tau PET, and MRI using five methods. For ^11^C-PIB (an amyloid PET radiotracer), a cutoff of 1.42 was defined based on a reliable worsening method, equivalent to 19 on the Centiloid scale. For FDG PET, tau PET, and MRI, different methods were applied with accuracy based on young clinically CU or age-matched clinically CU vs. cognitively impaired Aβ+ individuals. However, the cognitively unpaired Aβ+ group was selected based on the cutoff value of ^11^C-PIB (1.42).

The image-based cutoff values of Aβ biomarkers are diverse. The use of regional rather than global cutoff values could explain the variability in the results when evaluated with significant cognitive effects. It is attributed to certain subjects in the positive or negative groups with extremely focal Aβ deposition that may not be clinically meaningful in a group evaluation (Nebes et al., 2013). Recent studies (Landau et al., 2015; Farrell et al., 2018; Guo et al., 2020) have been focusing on the longitudinal evaluation of Aβ-CU individuals in specific brain regions, searching for regions of first Aβ accumulation and more indicative of a higher risk of cognitive decline. The use of regional cutoff values has enhanced the predicted memory decline, mainly when the most Aβ affected regions are used. The magnitude of Aβ change, not dichotomization, is a better predictor of risk for cognitive decline in Aβ-CU individuals (Farrell et al., 2017, 2018; Guo et al., 2020). The Centiloid method of scaling the Aβ burden is a better alternative for cutoff value variations on brain Aβ burden and staging of subjects. However, the cutoff values on the Centiloid scale to differentiate between normal loads of amyloid burden due to aging and disease are not yet completely known and require further studies.

## Biomarkers Joint Evaluation

According to Besson et al. (2015), the amyloid cascade consists of three stages for the preclinical phase of AD: (Peters, 2006) Aβ deposition alone, (2) Aβ deposition and neurodegeneration, and (3) Aβ deposition, neurodegeneration, and subtle decline. However, studies showed that neurodegeneration is not followed in this sequence or related to each other in a decline paradigm. In the NIA–AA research framework (Jack et al., 2018), the use of the AT(N) system is implemented to define the biomarker profile of amyloid and tau deposition and neurodegeneration and divided into categories. This system is classified on the basis of biomarkers, stages of normal AD biomarker, AD pathological change, AD and non-AD pathological changes, and independence of cognitive (clinical) status.

Tau pathology was found to be related to neurodegeneration as much as Aβ pathology, and NFTs can be observed in the aged brain even without the presence of Aβ plaques. NFTs are usually present around the medial temporal cortex, and Aβ presence expands these fibrillated taus to the entire cortex (Hanseeuw et al., 2017; Shimada et al., 2017; Maass et al., 2018). The anatomy of glucose hypometabolism correlated with the interaction between Aβ and neocortical tau distribution. Thus, hypometabolism in tau-associated regions may be an early imaging marker of memory decline in healthy elders with different levels of Aβ load (Hanseeuw et al., 2017). Imaging markers and the Aβ/tau ratio showed a predictive potential to decline in the Clinical Dementia Rating scale in healthy elders (Rizk-Jackson et al., 2013).

A combination of findings correlating with the presence of Aβ and tau showed that one potentiates physiological consequences of the other (Hanseeuw et al., 2017). It is still unclear whether Aβ pathology itself shows neurotoxicity *in vivo* and influences the clinical features (Shimada et al., 2017) or the mechanism or anatomic link that mediates Aβ and tau interaction (Hanseeuw et al., 2017). What has been reported is that the accumulation of Aβ and tau is associated with synaptic dysfunction and axonal degeneration and is correlated with changes in memory, global cognition, and axonal degeneration, which are useful for diseases prognosis (Pereira et al., 2021). The use of both amyloid and tau PET showed high potential as imaging markers of aging and cognitive decline. As tau radiotracers are still in development, more studies are required to evaluate the relationship between tau burden, neurodegeneration, and cognitive status.

## Open Questions

The review revealed that biomarkers are more complementary than the determinants. MRI, FDG PET, amyloid PET, and even tau PET show only one imaging marker above the normality threshold in healthy elders.

The majority of the reviewed studies comprised cross-sectional (and not longitudinal) data or only limited longitudinal information about the subjects. There still exists a lack of longitudinal studies exploring the relationship between images and aging markers. Moreover, the studies did not clarify the relationship between cognition, brain metabolism, and Aβ and/or tau accumulation in understanding dimensionality of the biomarkers in memory and cognitive decline.

Another issue identified is the lack of standardization of imaging markers. The Centiloid project has been trying to develop a standardized scale for the Aβ burden, using a well-delineated methodology for imaging analysis. The scale ranges from 0 for no Aβ burden in young, healthy adults to 100 for AD subjects with a high Aβ burden (Klunk et al., 2015). However, standardized methods for brain ^18^F-FDG PET, tau PET, and brain atrophy in MRI are still lacking. The cutoff values for positive and negative Aβ are still under discussion even with the Centiloid standardization. A composite biomarker is used to generate a new analysis approach, such as a combined ^18^F-FDG and MR biomarker for neurodegeneration or a composite score to determine the cognition spectrum.

The review showed the requirement for brain image patterns to identify the first signs of cognitive decline, enabling the implementation of new approaches for early therapeutic intervention. In addition, it emphasized the need for understanding the used biomarkers to detect the first changes leading to permanent cognitive decline and the possibility to intervene and differentiate dementia from other neurological diseases. In conclusion, we argue that in-depth studies on cognitive decline are required to understand it better and find the proper therapeutic intervention and its optimal windows for a suitable and effective treatment strategy.

## Author Contributions

CD contributed to study design, search, revision, writing, and discussion. WB critically reviewed and discussed the manuscript. MK critically reviewed the manuscript. AM contributed to study design and writing, and critically reviewed the manuscript. All authors approved the submitted version of the manuscript.

## Conflict of Interest

The authors declare that the research was conducted in the absence of any commercial or financial relationships that could be construed as a potential conflict of interest.

## Publisher's Note

All claims expressed in this article are solely those of the authors and do not necessarily represent those of their affiliated organizations, or those of the publisher, the editors and the reviewers. Any product that may be evaluated in this article, or claim that may be made by its manufacturer, is not guaranteed or endorsed by the publisher.
